# Morphological Aspects of Antennal Sensilla of the *Rhodnius brethesi* Matta, 1919 (Hemiptera: Reduviidae) from the Negro River, Amazon Region of Brazil

**DOI:** 10.1155/2020/7687041

**Published:** 2020-03-19

**Authors:** Simone Patrícia Carneiro Freitas, Laura Cristina Santos, Amanda Coutinho de Souza, Angela Cristina Verissimo Junqueira

**Affiliations:** ^1^Fundação Oswaldo Cruz-Piauí, Rua Magalhães Filho 519, Centro, Teresina, PI 64001-350, Brazil; ^2^Laboratório de Doenças Parasitárias, Instituto Oswaldo Cruz, FIOCRUZ, Av. Brasil 4365, Pavilhão Arthur Neiva, Sala 02, Rio de Janeiro, RJ 21040-360, Brazil

## Abstract

Studies conducted in river Ererê located in the left margin of Negro River, municipality of Barcelos, state of Amazonas, have confirmed that *Rhodnius brethesi* has as its natural habitat the palm tree *Leopoldinia piassaba*. By scanning electron microscopy, sensillum type was studied on the antennae of *R. brethesi*. The specimens used come from the field and laboratory colony. No differences were observed between *R. brethesi* and other Triatominae studied. In the *R. brethesi* antennas, differences were observed only between the antennal segments and in the dorsal and ventral portions. Trichobothria sensilla show a difference with a lamellar base, suggesting that this conformation of the base of the sensilla is a synapomorphic feature of the genus. Another important observation is that, considering that *R. brethesi* is a specialist, infesting only one type of palm tree, trichoidea sensilla may be involved with plant-derived odorants. The knowledge of such functions could benefit the understanding of the likely biological role of these structures in chemical communication and also provide basic information for future studies of niche recognition, since this species of triatomine is only found in the *L. piassaba* palm.

## 1. Introduction

Insect antennae are segmented appendages that are equipped with a variety of sensilla and function primarily as chemoreceptors, thermoreceptors, and hygroreceptors. Antennae play a crucial role in insect behavior, including host location and recognition, as well as mating behavior [1–3]. Sensilla may be structurally categorized by their external morphology, but structure alone is usually not sufficient to determine function. Another way of classifying these sensilla is based on a functional classification according to the stimuli the receptors are believed to respond to [1].

In Triatominae, certain sensilla (e.g., trichobothria) may have taxonomic value in Triatomine [4] whereas sensilla reflect selective pressure on insect habitat and hosts. As antennae are the main environmental sensors, the host species and habitat specialization of each species may be reflected by differences in their sensory characteristics, as suggested by mosquitoes [5, 6], beetles [7], and hymenopterans [8].

Triatomines perceive various stimuli through antennal sensory receptors [9]. The most important are chemoreceptors, which detect chemical components relating to food sources, sexual partner recognition, and habitat preferences [10–12]. Antennal phenotypes present differences that make it possible to distinguish triatomine genera, species, and even populations [12–15].

Our study is aimed at investigating and describing the morphology and the type of sensilla in the antenna of males and females of *Rhodnius brethesi* (Matta, 1919) (Hemiptera: Reduviidae) by scanning electron microscopy (SEM). This species is known as the “piassaba louse” among workers who extract fiber from the palm tree *Leopoldinia piassaba* Wallace (Arecales, Arecaceae) [16], which is an important economic activity in the upper and middle courses of the Rio Negro Brazilian Amazon region.

*Rhodnius brethesi* presents a particular danger to piassaba palm leaf collectors because palm trees are often infested with this species and they attack workers when they are sleeping in their huts locally in the forest [17, 18]. Studies in the Brazilian Amazon region showed that positive cases for *Trypanosoma cruzi* infection were attributed to the continuous exposure of workers to this vector species [19].

We speculated the functions of various sensilla and compared them with those that have been discussed based on morphology and ultrastructure. The knowledge of such functions could benefit the understanding of the likely biological role of these structures in chemical communication and also provide basic information for future studies of niche recognition, since this species of triatomine is only found in the *L. piassaba* palm.

## 2. Materials and Methods

The specimens used come from the field and laboratory colony. The specimens of the field were collected by means of modified Noireau traps [20] and Shannon-type traps on piassaba palm trees in river Ererê located in the left margin of Negro River, municipality of Barcelos, state of Amazonas, Brazil. The colony specimens were obtained from the 21^st^ generation of the *R. brethesi* collected in the left margin of Negro River, municipality of Barcelos, state of Amazonas, Brazil. The colonies are maintained in the Parasitic Disease Laboratory, Department of Tropical Medicine of Instituto Oswaldo Cruz.

Five males and five females of field specimens and five males and five females of specimens from the colony were used. The head was removed, washed in 70% alcohol, and mounted following the dorsal and ventral side of the antennas, on metallic supports suitable for scanning electron microscopy, using a double-sided tape. After being covered with a thin layer of gold, it was observed under a scanning electron microscope (JEOL JSM-6390; Jeol Corp., Tokyo, Japan) using the IOC Electron Microscopy Platform.

## 3. Results and Discussion

**Figure 1:**
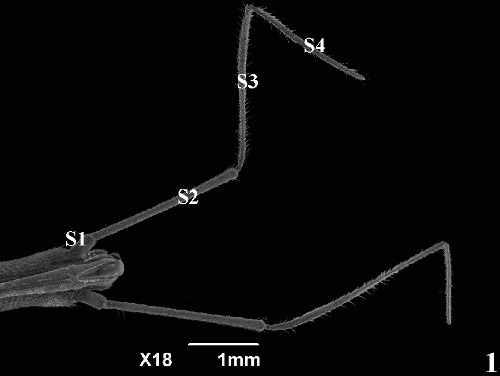
Scanning electron micrographs of the head of *R. brethesi* showing antennal segments. S1: first segment (scape); S2: second segment (pedicel); S3: third segment (flagellomere I); S4: fourth segment (flagellomere II).

Antennas of the *Rhodnius brethesi* have four segments (Figure 1) with the presence of sensilla of varying shapes and sizes. Analysis of antennal sensillum patterns showed that the types of sensilla present for *R. brethesi* were essentially the same morphological types described for the triatomines studied [9, 13, 21]. No differences in sensillum types were observed between males and females, be they field or colony. The differences were only observed between the antennal segments and in the dorsal and ventral portions.

**Figure 2:**
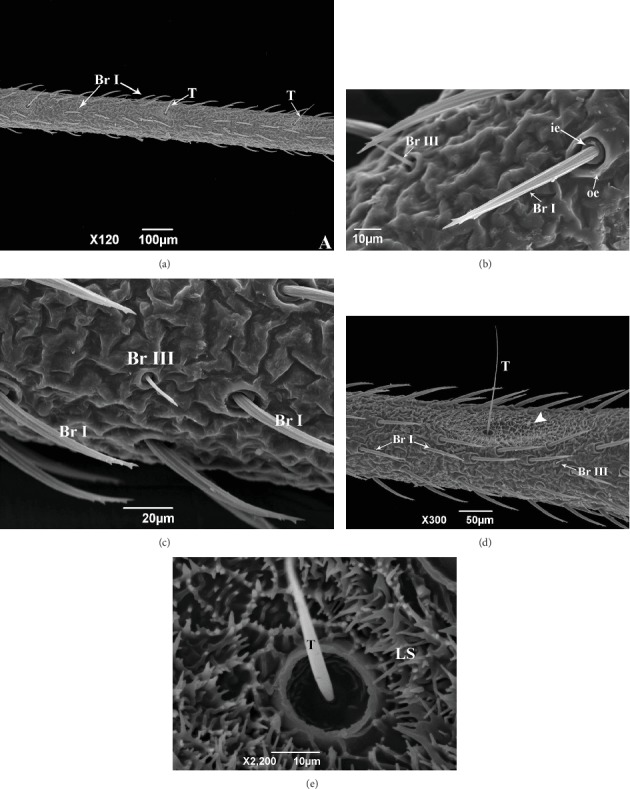
Scanning electron micrographs of the second antennal segment (S2) of *R. brethesi*. (a) Br I: bristles I; T: trichobothria. (b) Bristle I details. Br III: bristle III; ie: internal edge; oe: outer edge. (c) Br I: bristles I. Br III: bristle III. (d) Trichobothria sensilla (T) with a cuticular area at the base in the form of short extensions and lamellar structures (arrowhead). Br I: bristles I; Br III: bristle III. (e) Detail of the trichobothria sensilla (T), with a cuticular area at the base in the form of short extensions and lamellar structures (LS).

The second antennal segment (pedicel) of *R. brethesi* is covered by bristles I and Trichobothria sensilla (Figure 2(a)). Bristles I are characterized as thick bristles with prominent longitudinal grooves, ending in tubers (Figure 2(b)). Externally, two cuticular edges can be seen at the base of bristle I, being the thickest and highest outer edge (Figure 2(b)). In addition to this, we also see bristles III (Figure 2(c)), much shorter than bristles I and have double-ringed edge. Similar antennal sensilla were seen in *Triatoma infestans* (Klug, 1834) (Heteroptera: Reduviidae) and were impermeable to colorants, indicating a lack of pores. This and the described morphological characteristics suggest that these sensilla function as mechanoreceptors [22]. Bristles I and II are also commonly found in the insect nymph stage in *Triatoma* species [23].

Still in the second antennal segment, there is a row of trichobothria sensilla (Figure 2(a)) with a cuticular area at the base in the form of short extensions and lamellar structures (Figures 2(d) and 2(e)). These types of sensilla are already described for Triatominae [9, 12, 13, 21, 24, 25]. However, the latter are only seen in studies with *Rhodnius* species [9, 24], suggesting that lamellar-based trichobothria sensilla are a synapomorphic feature of the genus [4]. Another interesting observation, which was not mentioned in previous studies, is that these sensilla are only seen on the dorsal surface of the second antennal segment.

**Figure 3:**
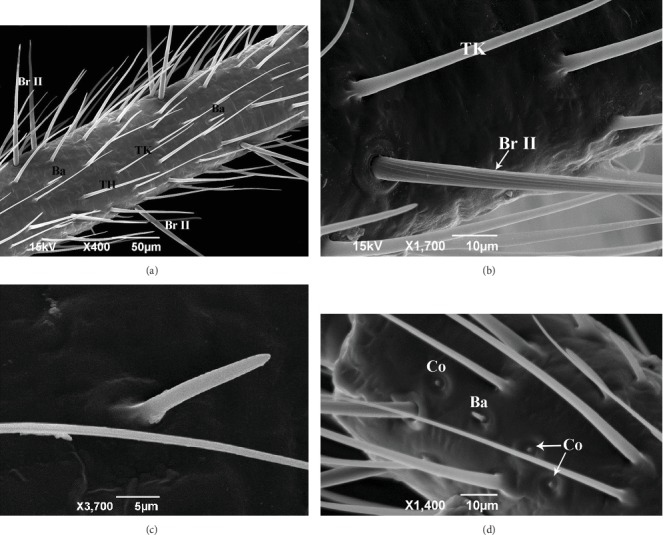
Scanning electron micrographs of the third antennal segment (S3) of *R. brethesi.* (a) Br II: bristles II; Ba: basiconica sensilla; TH: thin-walled trichoidea sensilla; TK: thick-walled trichoidea sensilla. (b) Bristles II (Br II) detail. TK: thick-walled trichoidea sensilla. (c) Basiconica sensilla. (d) Ba: basiconica sensilla; Co: coeloconica.

The third and fourth segments are fully lined with bristles II, thin-walled sensilla (thin-walled), thick-walled (thick-walled), basiconica (Figure 3(a)), and coeloconic (Figure 3(d)), all types already described for Triatominae [9, 12, 13, 21, 24, 25].

Bristles II are positioned on the sides of the body of the antennal segment (Figure 3(a)) and differ from bristles I of the second segment in being straighter and tapering to the tip. They carry longitudinal ridges similar to those of bristles I, but have no small tubers at the tip (Figure 3(b)). The thin-walled and thick-walled sensilla are similar to each other, but the thick-walled ones are slender and tapered to the tip (Figure 3(a)). In Triatominae, both types of trichoidea sensilla are found in the second antennal segment of the males and females; however, the number of sensilla showed the presence of sexual dimorphism. The males presented with a higher number of thin-walled sensilla and females with thick-walled sensilla [21].

Thin-walled trichoidea sensilla are strongly suggestive of an olfactory function. In *Triatoma infestans*, Mayer [26] demonstrated electrophysiological responses of these sensilla to human breath. In the present study, we did not consider the function of trichoidea sensilla, but considering that *R. brethesi* is a specialist, infesting only one type of palm tree [27], trichoidea sensilla may be involved with plant-derived odorants.

The short basiconica sensilla have a smooth surface and do not display a double-ringed edge (Figures 3(c) and 3(d)). On the first flagellar segment, the mean density of basiconica sensilla was similar in all *Rhodnius* species examined [9], but the mean density on the second antennal segment showed significant differences between species [9] and between females bred in the laboratory and wild females; the laboratory females presented lower number of basiconica sensilla, in comparison with wild females [21].

Coeloconic sensilla were found in the third and fourth antennal segments (Figure 3(d)). In all species of *Rhodnius* examined [9], these coeloconic sensilla are each innervated by three neurons with unbranched dendrites, and they are assumed to be thermohygroreceptors [28].

The third and fourth antennal segments are concentrated with the highest variability of sensillum types, with five types, while the second segment presents three types (Table 1). Sexual dimorphism in *R. brethesi* is marked by the density of sensilla [21]. In this study, in relation to morphological types of sensilla, the comparison between male and female antenna of *R. brethesi* did not show any sexual dimorphism among the specimens bred in the laboratory or field.

## Figures and Tables

**Table 1:** Characterization of each type of antennal sensilla according to the antennal segment in *Rhodnius brethesi*.

Sensillum type	Segments		
	S2	S3	S4
Bristles I	X		
Bristles II		X	X
Bristles III	X		
Basiconica		X	X
Coeloconic		X	X
Thick-walled		X	X
Thin-walled		X	X
Trichobothria	X		

## Data Availability

The main part of the data generated or analyzed during this study is included in this published article. Other data will be available from the corresponding author upon request.
